# Noscapine Prevents Rotenone-Induced Neurotoxicity: Involvement of Oxidative Stress, Neuroinflammation and Autophagy Pathways

**DOI:** 10.3390/molecules26154627

**Published:** 2021-07-30

**Authors:** Richard L. Jayaraj, Rami Beiram, Sheikh Azimullah, Nagoor Meeran M. F., Shreesh K. Ojha, Abdu Adem, Fakhreya Yousuf Jalal

**Affiliations:** 1 Department of Pharmacology and Therapeutics, College of Medicine and Health Sciences, United Arab Emirates University, Al Ain 17666, United Arab Emirates; richardlj@uaeu.ac.ae (R.L.J.); azim.sheikh@uaeu.ac.ae (S.A.); nagoormeeran1985@uaeu.ac.ae (N.M.M.F.); shreeshojha@uaeu.ac.ae (S.K.O.); 2 College of Medicine and Health Sciences, Khalifa University, Abu Dhabi 127788, United Arab Emirates

**Keywords:** Parkinson’s disease, noscapine, rotenone, oxidative stress, inflammation, autophagy

## Abstract

Parkinson’s disease is characterized by the loss of dopaminergic neurons in substantia nigra pars compacta (SNpc) and the resultant loss of dopamine in the striatum. Various studies have shown that oxidative stress and neuroinflammation plays a major role in PD progression. In addition, the autophagy lysosome pathway (ALP) plays an important role in the degradation of aggregated proteins, abnormal cytoplasmic organelles and proteins for intracellular homeostasis. Dysfunction of ALP results in the accumulation of α-synuclein and the loss of dopaminergic neurons in PD. Thus, modulating ALP is becoming an appealing therapeutic intervention. In our current study, we wanted to evaluate the neuroprotective potency of noscapine in a rotenone-induced PD rat model. Rats were administered rotenone injections (2.5 mg/kg, i.p.,) daily followed by noscapine (10 mg/kg, i.p.,) for four weeks. Noscapine, an iso-qinulinin alkaloid found naturally in the Papaveraceae family, has traditionally been used in the treatment of cancer, stroke and fibrosis. However, the neuroprotective potency of noscapine has not been analyzed. Our study showed that administration of noscapine decreased the upregulation of pro-inflammatory factors, oxidative stress, and α-synuclein expression with a significant increase in antioxidant enzymes. In addition, noscapine prevented rotenone-induced activation of microglia and astrocytes. These neuroprotective mechanisms resulted in a decrease in dopaminergic neuron loss in SNpc and neuronal fibers in the striatum. Further, noscapine administration enhanced the mTOR-mediated p70S6K pathway as well as inhibited apoptosis. In addition to these mechanisms, noscapine prevented a rotenone-mediated increase in lysosomal degradation, resulting in a decrease in α-synuclein aggregation. However, further studies are needed to further develop noscapine as a potential therapeutic candidate for PD treatment.

## 1. Introduction

Parkinson’s disease (PD) is the second most common neurodegenerative disease following Alzheimer’s disease. Clinical symptoms of PD include motor impairments, such as bradykinesia, rigidity, tremor and postural abnormalities. In addition, olfactory, cognitive and emotional anomalies are seen in PD patients [1]. Parkinson’s disease, the fastest growing neurodegenerative disease in prevalence and deaths doubled globally between 1995 and 2015 [2]. According to the global population ageing report, the ageing population of the United Arab Emirates has been projected to increase from 5.1% in 2000 to 26.7% by 2050. This might result in increase in the incidence of neurological disorders such as PD [3]. Various studies have reported that oxidative stress and neuroinflammation plays a major role in PD development and progression, which in turn influences: 1. mitochondrial dysfunction, 2. protein misfolding and 3. altered kinase activity [4]. Histopathological studies have shown that PD is characterized by the presence of Lewy bodies that are made up of intracellular alpha-synuclein inclusions [5]. Though PD is treated by dopamine receptor agonists, monoamine oxidase inhibitors and dopamine replacement therapy, the ability of these treatments to prevent the course of the disease is still limited. In addition, prolonged treatment causes adverse non-motor and motor side effects [6]. As mentioned earlier, neuroinflammation plays a major continuous role in dopaminergic neuronal loss that results in PD [7]. Neuroinflammation-mediated dopaminergic neuronal loss is driven by microgliosis and astrogliosis [8]. Various pro-inflammatory factors, such as ROS, TNF-α, IL-1β, COX-2 and iNOS, activate microglia that result in microglial morphological transformation and population expansion [9]. Similarly, astrocytes, the most populous glial subtype, play a key role in PD progression through reactive astrogliosis [10]. Reports demonstrate that alpha-synuclein deposits in the astrocytes initiate activation of microglia, which executes neurons, resulting in PD [11,12]. During the diseased state, reactive astrogliosis results in pro-inflammatory cytokine expression, ROS production, lipid peroxidation and apoptosis [10]. Recent studies have proved that administration of rotenone or MPP^+^, a mitochondrial complex I inhibitor, activates microglia and astrocytes, resulting in the production of pro-inflammatory factors (MMP-9, TNF-α, COX-2, iNOS) and subsequent neuronal death [13,14,15]. Though oxidative stress and neuroinflammation act as important initiators of PD, the most important cause of PD pathogenesis is impairment of misfolded protein clearance that results in the deposition of misfolded α-synuclein and impairment of protein degradation pathways that are toxic to dopaminergic neurons. In normal physiological conditions, autophagy plays a critical role in degrading misfolded proteins, non-specific long-lived proteins and impaired organelles to prevent apoptosis [16]. During autophagy, autophagosomes isolate misfolded proteins such as α-synuclein, which fuses with endosomes to form amphisomes. Further, amphisomes’ contents are degraded and recycled when amphisomes are fused with lysosomes [17,18]. However, studies have shown that autophagy-lysosome pathways are impaired in post-mortem PD samples, which leads to an accumulation of pathogenic α-synuclein in PD [19]. In addition, over-expression of mutant α-synuclein has been shown to activate autophagy [20,21]. Apart from ALP impairment, the loss of mammalian target of rapamycin (mTOR), an important protein for cell proliferation, growth and survival [22,23], has been reported in PD [24]. Similarly, hindering mTOR expression using siRNA interference prevents phosphorylation of p70S6 Kinase (p70S6K) and eukaryotic initiation factor 4E binding protein 1 (4E-BP1), resulting in apoptosis [25]. In addition, PD models such as N-methyl-4-phenylpyridine (MPP+), 6-hydroxydopamine (6-OHDA) and rotenone decreased mTOR signaling and up-regulated apoptotic proteins [26]. In contrast, restoration of mTOR, 4E-BP1 or p70S6K prevented dopaminergic neuronal loss in PD models [27,28].

Rotenone, a potent member of rotenoids and a classical inhibitor of mitochondrial complex I, is commonly used as a neurotoxin to understand the pathological mechanisms in PD. Distinct from MPP+, which requires specific dopamine transporters to induce dopaminergic neuronal death, rotenone, due to high lypophilicity, crosses the blood–brain barrier, effectively resulting in dopaminergic neuronal death [29]. Rotenone has been reported to cause oxidative stress, neuroinflammation, apoptosis of dopaminergic neurons, behavioral deficits, α-synuclein aggregation and impairment of ALP pathways [26].

Since PD is characterized by oxidative stress-mediated dopaminergic neurodegeneration, natural drugs with potent antioxidant and anti-inflammatory properties are used as the preferred treatment of choice due to fewer side effects [30]. However, apart from the antioxidative and anti-inflammatory properties, the mechanisms of neuroprotection of these natural drugs are still scarce. Hence, in this study, we aimed to understand the molecular neuroprotective mechanisms of noscapine in a rotenone PD model. Noscapine, a phthalideisoquinoline alkaloid from opium, has been reported to possess various biological properties [31]. Unlike other alkaloids obtained from opium, noscapine is devoid of analgesic, euphoriant or sedative effects. Hence, noscapine is extensively studied for possible treatment strategies for stroke [30]. In addition, noscapine has been reported to cross the blood–brain barrier, and therefore it has been studied for treatments of stroke, gliobastoma and anxiety [32,33,34]. To the best of our knowledge, this is the first study to report the neuroprotective mechanisms of noscapine in a rotenone-induced PD model. Our previous studies have shown that rotenone causes oxidative stress, neuroinflammation, α-synuclein expression and dopaminergic neuronal loss [13]. Hence, the rotenone rat model was used to evaluate whether neuroprotective mechanisms of noscapine are associated with mTOR-arbitrated anti-apoptotic and/or autophagic events.

## 2. Results

### 2.1. Noscapine Decreased Rotenone-Induced Oxidative Stress

**Figure 1:**
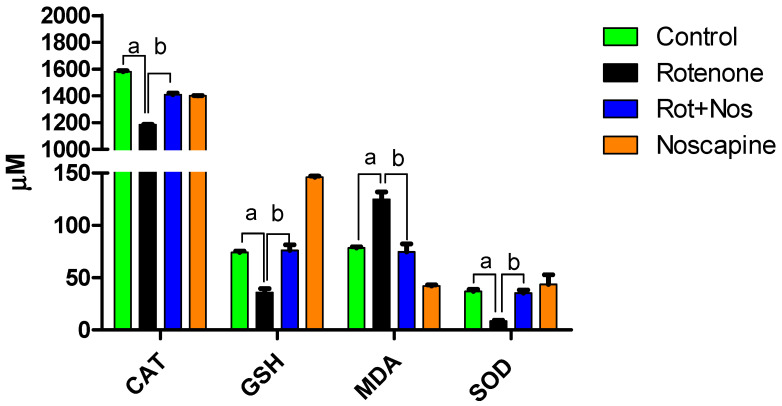
Noscapine prevented rotenone-induced oxidative stress and enhanced antioxidant enzymes in rats. Administration of rotenone increased (*p* < 0.05) lipid peroxidation, which is represented by increase in malonaldehyde levels with significant reduction in vital antioxidant levels in the midbrain (*n* = 5). However, treatment with noscapine prevented lipid peroxidation with a significant decrease in MDA levels and reverted brains’ vital antioxidant (CAT, GSH and SOD) levels. Data are expressed as mean ± SEM. ^a^ *p* < 0.05 compared to control, ^b^ *p* < 0.05 compared to rotenone-treated group.

Antioxidant enzymes are important in scavenging free radicals, a product of oxidative metabolism. We found that rotenone exposure induced a significant decrease in crucial antioxidants (Catalase, Glutathione and Superoxide dismutase) that play a major role in preventing free radical toxicity. Further, malondialdehyde (MDA) levels were significantly increased, which is a marker of lipid peroxidation. However, noscapine administration after rotenone exposure attenuated antioxidant enzyme loss associated with a decrease in MDA levels (Figure 1). Our results demonstrate that noscapine can prevent rotenone-induced oxidative stress in rats.

### 2.2. Noscapine Prevented Rotenone Mediated Neuroinflammation

**Figure 2:**
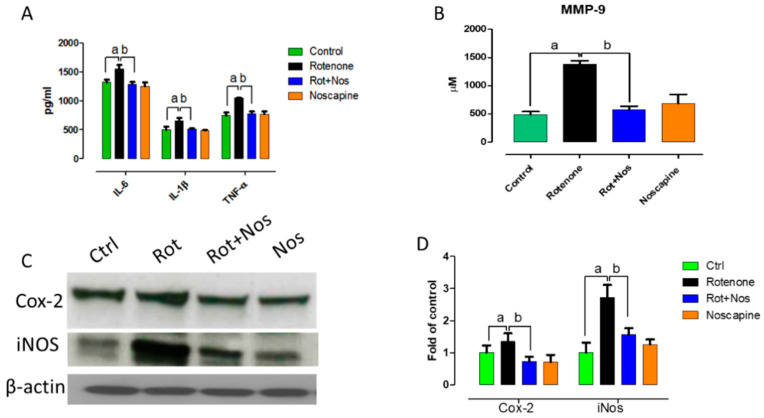
Noscapine inhibited MMP-9 production and modulated pro-inflammatory factors in experimental animals. Rotenone administration caused a significant increase in expression of pro-inflammatory cytokines (**A**) and enhanced production of MMP-9 (**B**). Western blotting analysis of midbrain protein samples probed with Cox-2 and iNOS (**C**). Quantitative analysis of western blots was performed using Image J and corresponding results were represented as bar diagram (**D**). However, noscapine treatment decreased expression and production of pro-inflammatory factors in rotenone-treated animals. Data are expressed as mean ± SEM. ^a^ *p* < 0.05 compared to control, ^b^ *p* < 0.05 compared to rotenone-treated group (*n* = 5).

Further, we wanted to evaluate the anti-inflammatory properties of noscapine. Hence, we examined the production and secretion of inflammatory factors upon noscapine treatment in a rotenone-induced PD model. Various reports show that pro-inflammatory factors augment apoptotic mechanisms. Our results showed that rotenone administration caused a significant increase in pro-inflammatory factors, as is evident from the increase in interleukin-6 (IL-6), interleukin-1β (IL-1β), tumor necrosis factor-α (TNF- α) and matrix metalloproteinase 9 (MMP-9) (Figure 2A,B). However, noscapine administration significantly decreased pro-inflammatory factors. In addition, rotenone administration enhanced the expression of inducible nitric oxide synthase (iNOS) and cyclooxygenase 2 (COX-2). It has been reported that over-expression of iNOS and COX-2 has been observed in the brains of PD patients, and this over-expression causes dopaminergic neuronal loss in PD models [35,36]. Supporting this evidence, our study showed that rotenone administration caused a significant increase in expression of iNOS and COX-2, whereas noscapine treatment significantly decreased these levels, as evidenced by Western blotting (Figure 2C,D).

### 2.3. Noscapine Prevented Activation of Microglia and Astroglia

**Figure 3:**
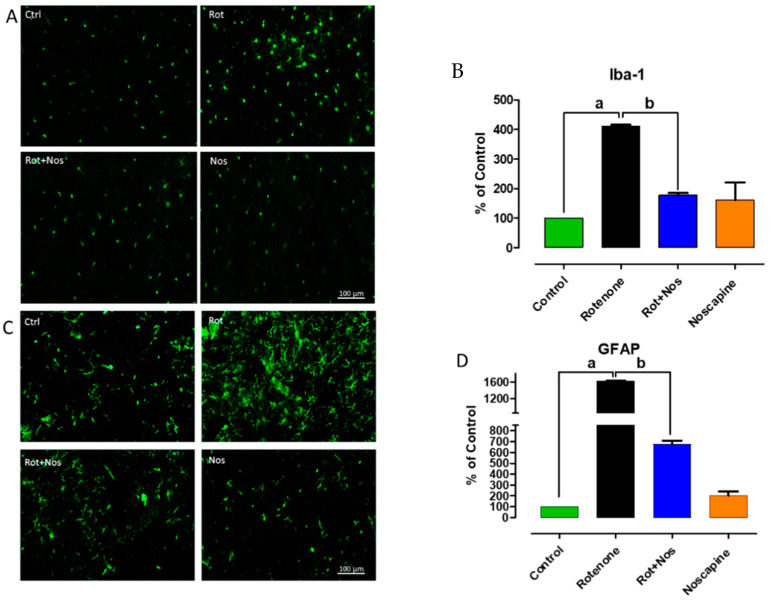
Noscapine prevented activation of microglia and astroglia. Enhanced expression of ionized calcium-binding adapter molecule 1 (Iba-1) and glial fibrillary acidic protein (GFAP) is an indicator of reactive microgliosis and reactive astrocytes, respectively. Our results (*n* = 5) showed that activation of microglia by rotenone is evident from larger cell bodies and fewer processes (**A**). Similarly, activation of astrocytes is evident from enhanced expression of GFAP positive cells (**C**). Conversely, administration of noscapine to rotenone-treated rats significantly decreased the expression of Iba-1 and GFAP in the striatum of experimental animals. These results were concordant with the decrease in production of pro-inflammatory factors in experimental animals. Quantification of activated microglia and astrocytes is represented as percentage of control and depicted as histogram in (**B**,**D**) respectively. Data are expressed as mean ± SEM. ^a^ *p* < 0.05 compared to control, ^b^ *p* < 0.05 compared to rotenone-treated group.

Over-expression of glial markers, especially ionized calcium-binding adapter molecule 1 (Iba-1) and glial fibrillary acidic protein (GFAP) gauge reactive microgliosis and reactive astrogliosis, respectively. Microglia and astrocytes fuel inflammation mediated neurodegeneration in PD. Immunofluorescent staining for Iba-1 and GFAP showed activation of microglia and astrocytes, respectively, in the striatum of rotenone-treated animals. Microglial activation is evident from larger cell bodies and fewer processes (Figure 3A). Similarly, astrocyte activation is confirmed by the enhanced expression of GFAP (Figure 3C). Interestingly, noscapine administration markedly reduced Iba-1 and GFAP expression in the striatum of experimental animals. These results were supported by a decrease in production of pro-inflammatory factors, as described previously. The quantification of activated microglia and astrocytes is represented as percentage of control and depicted as a histogram in Figure 3B,D, respectively.

### 2.4. Noscapine Protected Dopaminergic Neurons against Rotenone Neurotoxicity

**Figure 4:**
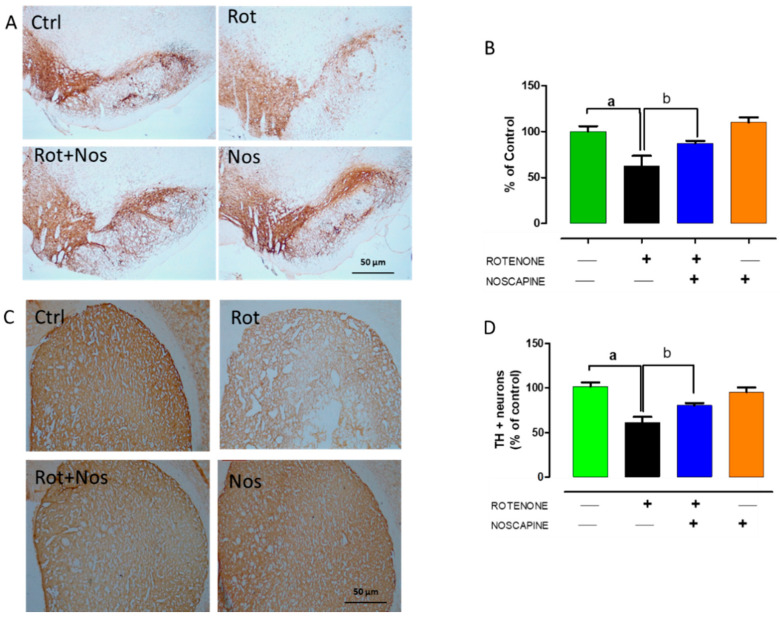
Noscapine protected dopaminergic neurons against rotenone toxicity. Parkinson’s disease is histologically characterized by significant loss of tyrosine hydrolase (TH)-positive dopaminergic neurons in the substantia nigra pars compacta (SNpc) with profound decrease in TH expression in the striatum. Therefore, we quantified TH-positive dopaminergic neurons in SNpc and expression of TH in the striatum (*n* = 5). Rotenone administration caused a significant decrease in number of TH positive neurons (**A**,**B**) which in turn caused significant decrease in the intensity of TH-positive striatal fibers (**C**,**D**). However, administration of noscapine prevented neuronal loss and increased TH expression in striatal fibers. Data are expressed as mean ± SEM. ^a^ *p* < 0.05 compared to control, ^b^ *p* < 0.05 compared to rotenone-treated group.

Loss of tyrosine hydrolase (TH)-positive dopaminergic neurons in SNpc and the subsequent decrease in TH expression in the striatum are hallmark histological events in PD. Thus, we evaluated TH-positive nigral dopaminergic neurons and their expression in the striatum. Rotenone administration significantly decreased the number of TH-positive neurons (Figure 4A,B), which in turn caused a 60% decrease in the intensity of TH-positive striatal fibers (Figure 4C,D). However, we found that noscapine administration prevented dopaminergic neuronal loss and enhanced TH expression in striatal fibers in rotenone-treated rats.

### 2.5. Noscapine Diminished Over-Expression of α-Synuclein in Experimental Rats

**Figure 5:**
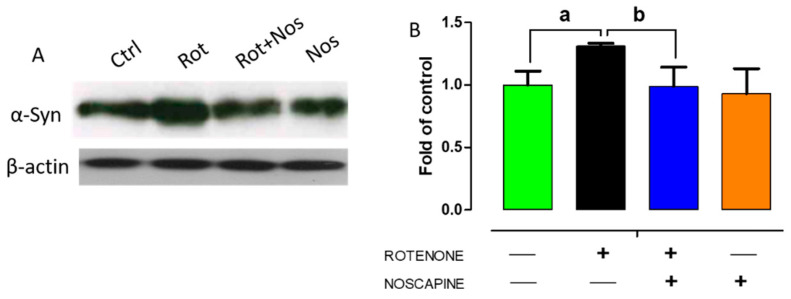
Noscapine modulates the expression of α-synuclein in rotenone-treated animals. Representative immunoblots of alpha-synuclein expression in experimental animals (*n* = 5) (**A**). Image J was used for quantitative analysis (**B**). Data are expressed as mean ± SEM. ^a^ *p* < 0.05 compared to control, ^b^ *p* < 0.05 compared to rotenone-treated group.

Alpha-synuclein aggregation results in Lewy body formation, a key pathological event in PD. Over-expression of α-synuclein enhances neuronal death by either necrosis or apoptosis [37]. In addition, autophagy impairment results in accumulation of aggregated α-synuclein, resulting in neurodegeneration. Our results showed that administration of rotenone significantly increased α-synuclein expression, as evidenced by Western blotting (Figure 5). In contrast, we found that noscapine treatment significantly decreased α-synuclein expression. These results demonstrate that noscapine partly prevents dopaminergic neuronal loss by decreasing the expression of α-synuclein in rotenone-treated animals.

### 2.6. Noscapine Prevented Neuronal Apoptosis by Restoring mTOR Pathway

**Figure 6:**
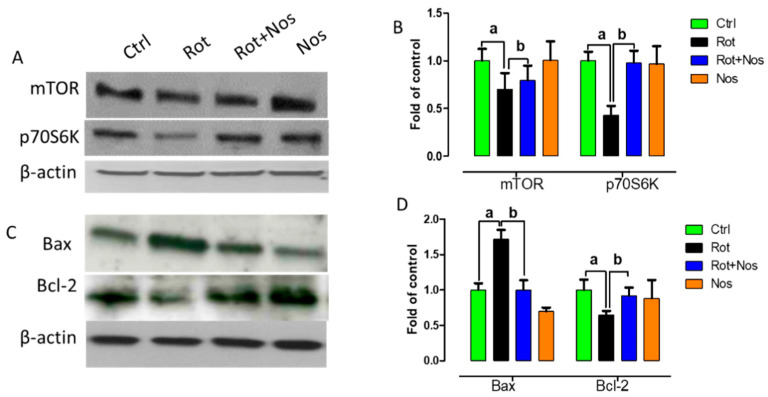
Noscapine diminished rotenone-induced neuronal apoptosis by restoring mTOR pathway. Western blotting of midbrain samples (*n* = 5) with mTOR pathway (**A**) proteins and apoptotic markers (**C**). Immunoblots of phosphor mTOR and p70S6K (**B**), Bax and Bcl-2 (**D**) were quantified using Image J and represented as fold of control. Results are expressed as mean ± SEM. ^a^ *p* < 0.05 compared to control, ^b^ *p* < 0.05 compared to rotenone-treated group.

Impairment of mTOR activity leads to neuronal dysfunction and prevents regenerative mechanisms [38,39]. mTOR regulates phosphorylation of p70 S6 kinase (p70S6K) and disruption of the mTOR pathway leads to apoptosis [40]. In the central nervous system, protein aggregation initially enhances mTOR expression, but its activity is subsequently reduced leading to cell death [41]. Hence, in this study, we wanted to analyze whether noscapine administration could prevent apoptosis by restoring mTOR activity. Western blot analysis demonstrated that rotenone significantly decreased mTOR and p70S6K expression (Figure 6A,B). However, noscapine treatment restored mTOR activity by increasing the expression of mTOR and p70S6K. Further, we wanted to evaluate the role of mTOR activity on neuronal apoptosis. Hence, we analyzed the levels of Bax and Bcl-2 by Western blotting. Rotenone administration enhanced the expression of pro-apoptotic protein Bax and diminished the expression of anti-apoptotic protein Bcl-2. In contrast, noscapine treatment hindered apoptosis by decreasing the expression of Bax and enhancing Bcl-2 expression (Figure 6C,D). These results demonstrate that noscapine prevented rotenone-mediated apoptosis by restoring the mTOR pathway.

### 2.7. Effect of Noscapine on Autophagy

**Figure 7:**
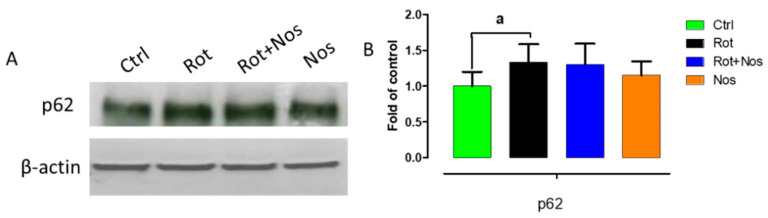
Noscapine prevented dopaminergic degeneration through regulating autophagy. Immunoblotting of midbrain samples (*n* = 5) with p62 (**A**). Quantitative analysis of immunoblotting of p62 (**B**). Values are represented as mean ± SEM. ^a^ *p* < 0.05 compared to control.

Autophagy impairment results in the accumulation of misfolded α-synuclein proteins and resultant neurodegeneration in PD. Hence, to further understand the neuroprotective mechanisms of noscapine, we evaluated whether noscapine could regulate rotenone-impaired autophagy by studying p62 protein expression levels (Figure 7A,B). P62 links ubiquitinated proteins to autophagic process through LC3. We found that rotenone administration significantly increased p62 levels, demonstrating rotenone-mediated autophagy impairment. However, we did not find a significant decrease in p62 levels after noscapine treatment.

## 3. Discussion

In the present study, the neuroprotective effect of noscapine was evaluated using a rotenone-induced PD rat model. We report for the first time that noscapine, a benzylisoquinoline alkaloid from opium, possesses anti-oxidative, anti-inflammatory, anti-apoptotic, anti-protein aggregation and autophagy modulating properties. In addition, noscapine protected dopaminergic neurons by restoring the mTOR pathway.

Rotenone, a classical mitochondrial complex I inhibitor is isolated from Leguminosa plants and is widely used in a PD rat model. Unlike MPP+, rotenone, being highly lipophilic, has the ability to cross the BBB and inhibits complex I of mitochondria, resulting in neuronal apoptosis. Our previous reports and studies by others demonstrate that rotenone-induced dopaminergic neurodegeneration involves the up-regulation of oxidative stress, behavior deficits, neuro-inflammation, α-synuclein aggregation, impairment of autophagy and nigrostriatal neuron loss [13,28,42]. Human post-mortem studies in PD also reported a marked decrease in antioxidant enzymes [43], inhibition of mitochondrial complex I [44] and enhanced oxidation/nitration of proteins and iron [45,46]. It is very well established that oxidative stress and redox imbalance are profound factors driving PD. Due to the limitations of synthetic drugs, natural phytobioactive compounds are gaining much attention due to their anti-oxidative and anti-inflammatory properties [47,48]. In addition, neuroprotective agents maintain neuronal integrity by neuroprotection, neuroplasticity and neurorestoration. Noscapine is one such neuroprotective agent that has the ability to cross the blood–brain barrier, which is an important characteristic for neuroprotective therapy [34]. Unlike other opium latex-based alkaloids, noscapine is devoid of sedative or analgesic properties [32]. Noscapine is suggested as an antitussive drug in several pharmcopeias [49]. Noscapine has been used as an antitussive agent in both animal models and in humans [50,51]. Though noscapine possesses various biological properties, the neuroprotective effect of noscapine in PD animal models has not yet been studied. Hence, we wanted to evaluate the antioxidant, anti-inflammatory and neuroprotective properties of noscapine in a rotenone-induced PD rat model. In our present study, rotenone treatment significantly decreased vital antioxidants GSH, CAT and SOD with a concomitant increase in lipid peroxidation, as demonstrated by high MDA levels. However, noscapine administration prevented free-radical production and lipid peroxidation. In the physiological state, monoamine oxidase inactivates dopamine, which results in significant production of hydrogen peroxide. Hence, higher dopamine turnover in early PD stages may generate higher hydrogen peroxide and subsequent hydroxyl radical production, resulting in dopaminergic neuronal death [52]. Therefore, noscapine, due to its anti-oxidative properties, could partly prevent neuronal death by scavenging free-radicals.

Several studies have reported that oxidative stress and neuroinflammation are associated with PD progression. Several clinical reports provide strong evidence that inflammation exacerbates neurodegeneration in PD [53]. Oxidative alternation of α-synuclein [54] and tyrosine hydrolase [55] precedes dopaminergic neurodegeneration. Therefore, it is possible that oxidants act as vital molecules activating neuroinflammation [43], impairment of autophagy systems and finally apoptosis of dopaminergic neurons [56]. In the CNS, reactive oxygen species have been largely produced by microglia due to oxidative mechanisms in mitochondria and intracellular peroxidases [57]. In line with previous studies, our results showed that rotenone-induced oxidative stress enhances microglia and astrocyte activation [58]. In PD, dopaminergic neurons die eventually by nonsynchronous events [59] and these events, though subtle, activate microglia and astrocytes, which results in the production of pro-inflammatory factors such as IL-6, IL-1β and TNF-α. Post-mortem studies have also shown up-regulated expression of pro-inflammatory factors in the brains of PD patients [60,61,62,63]. Similar to these findings, rotenone administration in our experimental rats hyper-activated microglia and astrocytes, which resulted in the over-expression of IL-6, IL-1β, TNF-α, MMP-9, COX-2 and iNOS. Nevertheless, noscapine administration prevented activation of microglia and astrocytes with a subsequent reduction in production of pro-inflammatory factors.

Alpha-synuclein plays a major role in enhancing neuroinflammation and it is an abundant protein in pre-synaptic terminals, which makes it prone to aggregation. Alpha-synuclein over-expression in a mouse model resulted in the activation of microglia with subsequent production of pro-inflammatory factors [64]. In addition, identification of alpha-synuclein in Lewy bodies of PD patients confirmed its pathological role in PD [65]. Hence, we wanted to evaluate the effect of noscapine administration in alpha-synuclein expression. Western blotting analysis showed that noscapine administration significantly decreased the expression of α-synuclein compared to rotenone-treated rats. Thus, a decrease in alpha-synuclein expression could be partially due to the anti-inflammatory properties of noscapine. Previous reports demonstrated that rotenone caused α-synuclein positive nigral inclusions and enhanced protein aggregation that resulted in neuronal death [66]. Further, to understand whether these biological properties of noscapine could protect dopaminergic neurons from apoptosis, we analyzed tyrosine hydrolase expression in substantia nigra and striata of experimental animals. We found that rotenone administration significantly depleted dopaminergic neurons in SN with a subsequent decrease in tyrosine hydrolase expression in striatal fibers. Our results were in line with our previously published report [67]. However, noscapine administration prevented dopaminergic neuronal loss and striatal fiber loss in rotenone treated animals as evidenced by TH immunohistochemistry. Further, we wanted to evaluate the molecular pathways involved in the neuroprotection offered by noscapine.

Apoptosis, an important pathway for programmed cell death, plays a major role in dopaminergic neurodegeneration. Apoptosis is characterized by morphological and biological changes such as cell shrinkage, chromatin condensation, DNA fragmentation and the development of apoptotic bodies which are eventually removed by phagocytosis without inflammatory stimuli. Various reports demonstrate the presence of apoptotic neurons in substantia nigra of PD patients [68,69]. In addition, wild-type or mutant forms of α-synuclein result in apoptosis and enhances neuronal sensitivity to apoptotic death [70]. The intrinsic apoptotic pathway involves the release of cytochrome c into the cytosol, membrane permeabilization by pore-forming protein Bax and a decrease in anti-apoptotic protein Bcl-2 that results in diminished ATP synthesis. In addition, the enhanced expression of alpha-synuclein suppresses the expression of anti-apoptotic protein Bcl-2 [71]. Similarly, in an MPTP PD model, apoptosis is characterized by ROS production, cytochrome C release, DNA fragmentation and other morphological features of apoptosis [72]. Zhou et al. (2015) recently reported that the blockage of mTOR signaling intensifies rotenone-mediated neuronal apoptosis [27]. The mTOR signaling has a crucial role in the maintenance of cell shape, migration and differentiation during neuronal development [73]. mTOR signaling has also been reported to be crucial for memory formation [74,75] and synaptic plasticity [27]. In addition, the suppression of mTOR-associated p70S6K and 4E-BP1 stimulates apoptosis [22]. The administration of rotenone enhances apoptosis of neurons by suppressing the mTOR cascade, suggesting the neuroprotective significance of the mTOR pathway [27]. In line with these reports, our results showed that rotenone administration suppressed the expression of mTOR and p70S6K and triggered apoptosis by decreasing the expression of Bcl-2 and enhancing Bax expression. However, noscapine restored the mTOR pathway by enhancing the expression of mTOR and p70S6K and prevented apoptosis. These results suggest that noscapine partly prevents dopaminergic neurodegeneration through its anti-apoptotic property.

The impairment of autophagy, an important catabolic mechanism involved in degradation of misfolded proteins, represents an important pathobiological event in PD. Various reports provide strong evidence that the build-up of autophagic vacuoles is toxic to dopaminergic neurons [69,76,77]. Under normal physiological conditions, heat shock cognate 70 (HSC70) chaperone protein binds to α-synuclein by identifying the pentapeptide sequence motif [78]. Later, α-synuclein binds to lysosomal-associated membrane protein type 2A (LAMP-2A) at the lysosomal membrane, where it is degraded by proteases. Apart from the protective mechanisms, autophagy has been acknowledged to be related to PD [79]. Post-mortem studies reported that autophagosomes containing neuromelanin and lipofuscin are present in degenerating neurons [80]. Rodent studies showed that rotenone administration resulted in an accumulation of autophagic vacuoles, as evidenced by enhanced LC3 expression. Further, autophagic vacuole accumulation was correlated with a decrease in lysosomal degradation, as is evident with the increase in p62 levels (autophagy substrate) [81]. Hence, we assessed p62 levels, which are usually a marker for autophagic flux. P62 recognizes ubiquitinated proteins and LC3 binding protein that binds with aggregated proteins, resulting in autophagy degradation. However, rotenone treatment enhances p62 levels, indicating that rotenone blocks autophagic vacuole degradation [81]. In our study, noscapine administration tends to decrease p62 levels, although this was not significant. Similar to our reports, Magalhaes et al. (2018) reported that ambroxol, a GCase chaperone, decreased LC3 expression and enhanced p62 levels [82]. To conclude, similarly to previous reports, rotenone enhances autophagic vacuole accumulation and ruins autophagic flux [81]. In addition, mTOR is a negative regulator of autophagy [83,84]. Hence, our data suggest that noscapine protects dopaminergic neurons by reducing the α-synuclein expression and activating the mTOR pathway, resulting in less autophagic vacuole formation and restored autophagy flux.

## 4. Materials and Methods

### 4.1. Experimental Animals and Ethics Statement

Male Wistar rats (280–300 g) were used in this experiment and the Animal Ethics Committee of United Arab Emirates University (UAEU) approved all the animal procedures. Throughout the study, rats were maintained at ambient conditions (22 ± 1 °C, 60% humidity and 12 h diurnal cycle) and rats had ad libitum access to water and food. One week prior to animal injections, rats were allowed to adapt to the experimental room conditions.

### 4.2. Chemicals and Reagents

Noscapine, RIPA lysis buffer, rotenone and antibodies (inducible nitric oxide synthase (iNOS), cyclooxygenase-2 (Cox-2) and glial fibrillary acidic protein (GFAP)) were purchased from Sigma-Aldrich, St. Louis, MO, USA. Anti-tyrosine hydrolase (Polyclonal rabbit) antibody was obtained from Merck, Germany. Protease and the phosphatase-inhibitor cocktail were purchased from Thermo Scientific, Rockford, IL, USA. The following antibodies were purchased from Cell Signaling Technology, Beverly, MA, USA: p62, mTOR and p70S6K. Apoptotic polyclonal markers (Bax and Bcl-2) were obtained from Abcam, Cambridge, MA, USA. Monoclonal mouse anti-α-synuclein antibody was procured from BD Biosciences, San Jose, CA, USA. Anti- Iba-1 antibody was purchased from Wako chemicals, Richmond, VA, USA. Alexa Flour 488 fluorescent secondary antibodies were obtained from Thermo Fischer Scientific, Waltham, Massachusetts, USA. Goat biotinylated anti-rabbit secondary antibody was obtained from Jackson Immunoresearch, West Grove, PA, USA. All biochemical assays were completed using commercially available kits. All other chemicals used in these experiments were analytical-grade quality procured from local commercial sources.

### 4.3. Experimental Design

The chronic rotenone paradigm, which represents one of the most stable toxin-based PD models, was applied to analyze the neuroprotective effect of noscapine. Rotenone (2.5 mg/kg) preparation was performed according to our previously published protocol [13,67]. The neuroprotective effects of noscapine in vivo were studied using the following treatment groups (*n* = 15/group). A control group received an intraperitoneal injection of myglol and olive oil, which are vehicles for rotenone and noscapine, respectively, which served as the control group. A rotenone group (experimental PD model) received rotenone (2.5 mg/kg, i.p.,) once daily for 4 weeks. A rotenone+noscapine group received noscapine (10 mg/kg, i.p.,) once daily for four weeks, 30 min prior to ROT administration. A noscapine-only group received an intraperitoneal injection of noscapine (10 mg/kg) once daily for four weeks and served as the drug control.

### 4.4. Tissue Processing

Animals were anesthetized using pentobarbital (40 mg/kg body weight) at the end of the experiment and perfused via infusion (intracardial) with 0.01 M phosphate-buffered saline at pH 7.4. After infusion with 0.01 M PBS, animals were again perfused with 4% paraformaldehyde for whole-body fixation for immunohistochemical studies. Their brains were carefully and post-fixed with 4% paraformaldehyde for 48 h (4 °C), followed by cryoprotection with 30% sucrose solution for 3 days (4 °C). The midbrain and striatum for biochemical studies were dissected on dry ice and immediately homogenized using KCl buffer (Tris–HCl 10 mM, NaCl 140 mM, KCl 300 mM, ethylenediaminetetraacetic acid 1 mM, Triton-X 100 0.5%) at pH 8.0, supplemented with protease and phosphatase inhibitor. The resulting homogenates were centrifuged at 14,000× *g* for 20 min (4 °C). The supernatant was used for lipid peroxidation, antioxidant enzymes and proinflammatory cytokine analysis using spectrophotometric measurements and enzyme-linked immunosorbent assays (ELISA).

### 4.5. Malondialdehyde Assay

A malondialdehyde assay was performed to understand lipid peroxidation in the midbrain of experimental animals using an MDA detection kit (North West Life Science, Vancouver, WA, USA). Initially, 250 μL of calibrators or samples were incubated with thiobarbituric acid, and this mixture was vortexed vigorously. One hour after incubation at 60 °C, the mixture was centrifuged (10,000× *g*, 3 min). The reaction mixture was transferred to cuvette and the spectra were measured at 532 nm. The results were expressed as μm MDA/mg protein.

### 4.6. Quantification of Reduced Glutathione

Sigma’s glutathione assay kit (Sigma-Aldrich Chemie GmbH, Steinheim) was used to analyze reduced glutathione levels in tissue homogenates, as described previously [65]. In short, brain samples were deproteinized with 5% 5-sulfosalicylic acid solution and then centrifuged to remove the precipitated protein. The resulting supernatant was used to analyze GSH content in the sample. Standards or samples (10 μL) were incubated with 150 μL of working mixture (assay buffer +5,5′-dithiobis (2-nitrobenzoic acid) + GSH reductase) in 96-well plates. After 5 min, diluted NADPH solution (50 µL) was added into each well and mixed thoroughly. Absorbance was measured at 412 nm with the kinetics for 5 min by using the microplate reader. Results were expressed as μm GSH/mg protein.

### 4.7. Assay for Antioxidant Enzyme Activities

Superoxide dismutase (SOD) and catalase (CAT) levels in the midbrain were measured using Cayman assay kits (Cayman Chemicals Company, Ann Arbor, MI, USA). Briefly, for SOD measurements, ten microliters of standards or samples were added into 96-well plates. Xanthine oxidase (20 μL) was added to the standard and samples to initiate the reaction. After mixing the reaction mixture for few seconds, the mixture (covered) was incubated at room temperature for 30 min. Absorbance was read at 450 nm using a microplate reader. The activity of SOD was expressed as units/mg protein.

Catalase assay: Similarly, samples or standards (20 μL) and 30 μL of methanol was added to the assay buffer (100 μL) in 96-well plates. Hydrogen peroxide (20 μL) was added to this mixture and incubated for at room temperature (RT) to initiate the reaction. After 20 min, 30 μL of potassium hydroxide was added to stop the reaction, followed by the subsequent addition of catalase purpald (30 μL) and catalase potassium periodate (10 μL). The resulting mixture was incubated at room temperature for 5 min in a shaker and the plate was read at 540 nm using a microplate reader. Catalase activity was expressed as nmol/min/mg protein.

### 4.8. Proinflammatory Cytokines and MMP-9 ELISA Assay

The secretion of pro-inflammatory cytokines (TNF-α, IL-1β, IL-6) and MMP-9 was assessed using commercially available ELISA kits (BioSource International Inc., Camarillo, CA, USA). In short, 96-well plates were coated with capture antibody (100 μL, diluted) and incubated at room temperature overnight. Later, the plate was washed using wash buffer (0.05% Tween 20 in PBS 0.01 M pH 7.4) and blocked for 1 h with blocking buffer (1% bovine serum albumin in PBS (300 μL)). The plates were then washed with washing buffer to remove excess blocking buffer, and samples or standards (100 μL) were added into each well. The plate was incubated for 2 h at RT. After incubation, the detection antibody (100 μL) was added into each well and the plate was incubated for 2 h at room temperature. The plates were later washed with wash buffer and 100 μL of working solution (1:200, streptavidin horseradish peroxidase) was added and incubated for 20 min at room temperature. After incubation, 100 μL of substrate solution was added and the plate was again incubated for 20 min. Finally, stop solution (2N H_2_SO_4_, (50 μL)) was added onto the plate and the contents in the plate were mixed by gentle tapping. Absorbance was read immediately at 450 nm using a microplate reader. The results were expressed as pg/mg protein.

### 4.9. Assessment of Microglia and Astrocyte Activation by Immunofluorescence Staining

The effect of noscapine on microglia and astrocyte was studied using immunofluorescent staining. Briefly, 20 μm striatal sections were washed gently with PBS and the sections were incubated with blocking reagent (10% normal goat serum in PBS 0.3% Triton-X 100) at room temperature. After 1 h, anti-rabbit Iba-1 (1:1000) and anti-rabbit GFAP (1:1000) antibodies was added and the sections were incubated overnight at 4 °C. The sections were then washed twice with PBS, and the corresponding fluorescent secondary antibody (Alexa 488 anti-rabbit) was added and incubated for 1 h at RT. The stained sections were again washed twice with PBS and mounted using Vectastain fluorescent mounting media (with DAPI). The images were taken under a Nikon Eclipse Ni fluorescent microscope.

### 4.10. Quantification of Activated Astrocytes and Microglia in the Striatum

A minimum of three coronal sections of similar levels of striata from each brain were used to analyze the morphology and activation of astrocytes and microglia, according to our previously published protocol [13,85]. Three different fields of equal area were chosen randomly, and the fluorescence intensity was analyzed using the Image J software (NIH, Bethesda, MD, USA). Briefly, an outline was drawn around the region of interest and area, circularity and mean fluorescence were measured, along with several adjacent background readings. The total corrected cellular fluorescence (TCCF) was calculated using the formula, TCCF = integrated density (area of selected cell × mean fluorescence of background readings). All measurements were performed by an observer blind to the treatment conditions to prevent bias. Results were represented as percentage of control.

### 4.11. Immunoblot Analysis

Western blot analysis was performed to understand the expression of α-synuclein, COX-2, iNOS, Bax, Bcl-2, mTOR, p62 and p70S6K in the striatum of experimental animals [65]. Briefly, striatal tissues were homogenized in RIPA buffer (with protease and phosphatase inhibitor), and the samples were centrifuged at 12,000 rpm for 20 min to obtain the supernatant. Protein (20 µg) from each sample was loaded and separated using SDS-PAGE. Separated proteins were then electrotransferred onto a PVDF membrane by the semi-dry transfer method (BIO-RAD). Later, the membranes were blocked with blocking solution (1 h in 5% nonfat dry milk in TBS at RT) and incubated overnight at 40 °C with corresponding primary antibodies (α-synuclein (1:750), COX-2 (1:2000), iNOS (1:1000), Bax (1:2000), Bcl-2 (1:500), mTOR (1:1500), p62 (1:900) and p70S6K (1:900)). Later, membranes were washed and then incubated for 1 h at room temperature with horseradish peroxidase-conjugated secondary antibodies. The membranes were washed to remove unbound secondary antibodies and the bands were visualized using an Enhanced Chemiluminescence Pico Kit (Thermo Fisher Scientific). The blots were stripped and re-probed for β-actin (1:5000; monoclonal mouse; EMD Millipore, Billerica, MA, USA) as a loading control and densitometry analysis was performed using “Image J” analysis software.

### 4.12. Immunohistochemistry Analysis

Cryoprotected rat brain sections (20 μm) were washed twice with 0.01 M of PBS, pH 7.4, and incubated for no longer than 10 min with 1% hydrogen peroxidase (in PBS) to inactivate tissue peroxidase. After washing twice with PBS, the sections were incubated using a blocking reagent (10% normal goat serum in PBS containing 0.3% Triton-X 100) for 30 min at RT. After blocking, goat anti-rabbit tyrosine polyclonal antibody (1:1000) was added and the sections were incubated overnight at 40 °C. Sections were then rinsed twice with PBS and biotinylated secondary anti-rabbit (1:1000) antibody was added to the sections and incubated for 1 h at room temperature. After washing to remove unbound secondary antibodies, sections were developed using the avidin-biotin peroxidase complex system (ABC kit, Vectastain, CA, USA) followed by 3,3′ diaminobenzidine (DAB) to analyze TH immunoreactivity. Stained sections were coverslipped using DPX mounting medium, and the slides were viewed under a light microscope (Olympus, Hamburg, Germany).

### 4.13. Assessment of TH-ir Dopaminergic Neurons and TH-ir Dopamine Nerve Fibers Loss

The effect of noscapine on TH-immunoreactive (TH-ir) neurons in the SNc area was assessed by counting three different levels of the medial terminal nucleus region, and the average value was represented as percentage of control [65]. Loss of striatal fiber was assessed by analyzing the optical density of TH-ir dopaminergic fibers in the striatum using Image J software. Briefly, the optical density of TH-ir fibers at three different fields from each section with an equal area within the striatum was measured for each rat, and the average of the three areas was calculated and depicted as percentage of control. For background measurements, the optical density of the overlying cortex was taken and subtracted from the value generated from the striatum. The counting of TH-ir neurons and optical density of the TH-ir fibers was carried out by an observer blind to the experimental groups.

### 4.14. Protein Estimation

Pierce BCA protein assay kit (Thermo Fisher Scientific) was used to quantify the protein concentration in each sample following the manufacturer’s instructions.

### 4.15. Statistics

All experimental data were expressed as mean value ± SEM. Statistical significance between various groups was assessed using one-way analysis of variance followed by Tukey’s test using SPSS 12 software. In all the experiments, *p* < 0.05 was considered statistically significant.

## 5. Conclusions

In summary, our results showed that noscapine protected dopaminergic neurons by (i) suppressing rotenone induced oxidative stress by restoring antioxidant enzyme activities, (ii) blocking inflammation by suppressing the activity of microglia and astrocytes and the subsequent production of pro-inflammatory factors, and (iii) regulated the autophagy pathway by inhibiting autophagy vacuole accumulation and promoted lysosomal degradation of α-synuclein. Therefore, noscapine could be further developed as a therapeutic candidate for PD.

## Sample Availability

Noscapine is purchased commercially from Sigma.

## Abbreviations

PD	Parkinson’s disease
ROS	Reactive oxygen species
GFAP	Glial fibrillary acidic protein
Cox-2	Cyclooxygenase 2
iNOS	Inducible nitric oxide synthase
Rot	Rotenone
SOD	Superoxide dismutase
CAT	Catalase
TNF-α	Tumor necrosis factor-alpha
IL-1β	Interleukin-1β
Iba-1	Ionized calcium-binding adapter molecule 1
TH	Tyrosine hydrolase
LC3B	Microtubule-associated protein 1 light chain 3 beta
mTOR	Mammalian target of rapamycin
4E-BP1	Eukaryotic translation initiation factor 4E (eIF4E)-binding protein 1
